# Soluble Urokinase-Type Plasminogen Activator Receptor and Arterial Stiffness in Patients with COPD

**DOI:** 10.1007/s00408-019-00211-w

**Published:** 2019-02-28

**Authors:** Renáta M. Böcskei, Béla Benczúr, György Losonczy, Miklós Illyés, Attila Cziráki, Veronika Müller, Anikó Bohács, András Bikov

**Affiliations:** 1 Department of Pulmonology, Semmelweis University, Diós árok Street. 1/c, Budapest, 1125 Hungary; 2 1st Dept of Internal Medicine (Cardiology/Nephrology), Balassa Janos County Hospital, Béri Balogh Ádám Street 5-7, Szekszárd, 7100 Hungary; 3 Heart Institute, Faculty of Medicine, University of Pécs, Ifjúság Street 13, Pecs, 7624 Hungary

**Keywords:** COPD, SuPAR, IL-6, Arterial stiffness, Cardiovascular risk

## Abstract

**Introduction:**

Soluble urokinase-type plasminogen activator receptor (suPAR) is upregulated by inflammation and plays a role in the pathogenesis of atherosclerosis. Chronic obstructive pulmonary disease (COPD) is associated with enhanced systemic inflammation and increased risk for atherosclerosis, however, studies analysing the circulating suPAR levels in COPD are contradictory. The aim of the study was to investigate plasma suPAR concentrations together with markers of arterial stiffness in COPD.

**Materials and Methods:**

Twenty-four patients with COPD and 18 non-COPD, control subjects participated in the study. Plasma suPAR was measured, together with lung volumes, symptom burden, exacerbation history, markers of arterial stiffness and soluble inflammatory biomarkers, such as endothelin-1, high-sensitivity C-reactive protein (hsCRP), interleukin-6 (IL-6).

**Results:**

Plasma suPAR levels were higher in COPD (2.84 ± 0.67 ng/ml vs. 2.41 ± 0.57 ng/ml, *p* = 0.03) and were related to lung function measured with FEV_1_ (*r* = − 0.65, *p* < 0.01) and symptom burden determined with the modified Medical Research Council questionnaire (*r* = 0.55, *p* < 0.05). Plasma suPAR concentrations correlated with various measures of arterial stiffness in all subjects, but only with ejection duration in COPD (*r* = − 0.44, *p* = 0.03).

**Conclusions:**

Plasma suPAR levels are elevated in COPD and relate to arterial stiffness. Our results suggest that suPAR may be a potential link between COPD and atherosclerosis.

## Introduction

Chronic obstructive pulmonary disease (COPD) is a chronic, progressive disorder of the airways and lung parenchyma and is one of the leading causes of mortality worldwide [1]. Chronic exposure to noxious particles, especially to smoking induces airway inflammation which eventually leads to mucus hypersecretion, emphysema and small airway narrowing [2]. COPD is also associated with small-grade systemic inflammation which may be a potential link to cardiovascular comorbidities, such as atherosclerosis [3–5]. However, the elements of systemic inflammation are poorly described in COPD.

Soluble urokinase-type plasminogen activator receptor (suPAR) is a soluble form of the urokinase plasminogen activator receptor (uPAR) that is produced upon cleavage of membrane-bound uPAR. It is found in various body fluids, including blood, urine and cerebrospinal fluid [6–8]. It is expressed by endothelial cells, macrophages, monocytes, neutrophils, lymphocytes and fibroblasts [9], and is upregulated by infections and pro-inflammatory cytokines [6]. The suPAR contributes to plasminogen activation, cell adhesion, chemotaxis and immune cell activation [10]. Clinical studies suggest that suPAR has an additive value to high-sensitivity C-reactive protein (hsCRP) or interleukin-6 (IL-6) in characterizing systemic inflammation in cardiovascular diseases [11].

So far, only few studies have investigated suPAR in COPD, mainly focusing on acute exacerbations and reporting elevated levels [12, 13]. In stable disease, Can et al. reported higher serum suPAR levels compared to controls [14]. This contradicts a study by Wang et al. who did not find significant difference between COPD and those in a healthy condition [15]. The discrepancy may be due to the relatively mild disease severity of the latter study [15], as the former study assessed patients with a wider range of lung function [14]. In addition, a number of factors which characterize disease burden apart from lung function, including symptom burden, exacerbation history and arterial stiffness, has not been assessed.

The aim of the present study was, therefore, to compare circulating suPAR levels in COPD and health, and to correlate them with various characteristics of COPD. To study suPAR in the context of other inflammatory biomarkers we also analysed hsCRP, IL-6 and endothelin-1.

## Materials and Methods

### Study subjects

**Fig. 1:**
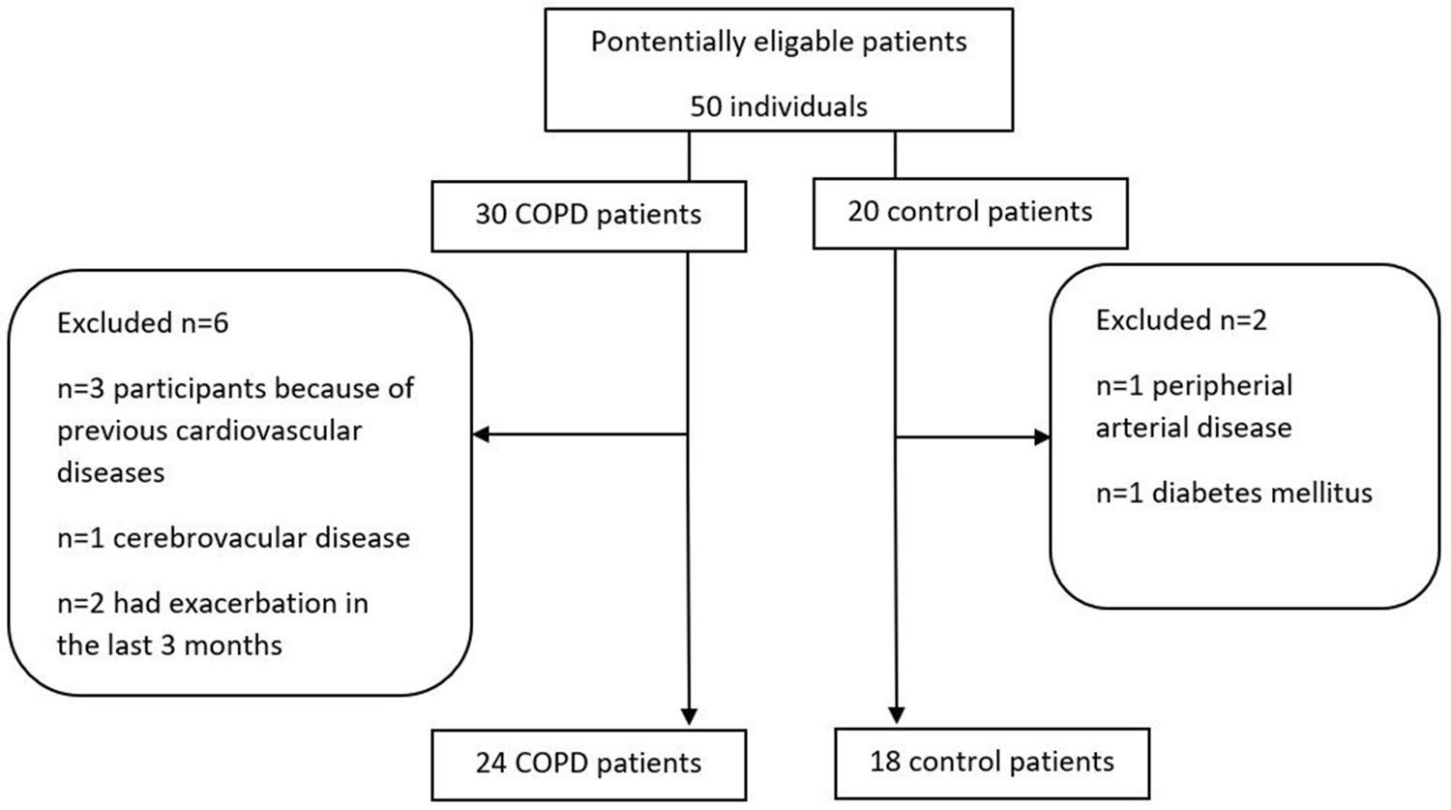
Flow chart of the number and selection of individuals in the study population

A total of 42 middle aged individuals (*n* = 19 males, mean age: 59 ± 11 years), were included in the study. Figure 1 displays the enrolment of participants into the study. COPD patients (*n* = 24) were recruited at stable state at the outpatient clinic of the Department of Pulmonology, Semmelweis University. COPD was diagnosed according to the Global Initiative for Chronic Obstructive Lung Disease (GOLD) criteria based on symptoms, suggestive history and post-bronchodilator FEV_1_/FVC < 0.70 [1]. Patients were categorised into ABCD subgroups according to the 2017 GOLD criteria [1]. Exacerbations in the last 12 months were defined as episodes requiring an increase in inhaler use or need for addition of antibiotics and/or systemic steroids. Frequent exacerbator phenotype was defined as having ≥ 2 exacerbations last year. None of the patients had suffered from acute exacerbation in the last 3 months. Control volunteers (*n* = 18) were recruited among co-workers at the Department of Pulmonology. Subjects with known cardiovascular disease, including coronary artery disease, cerebrovascular disease, or peripheral arterial disease and those who had diabetes mellitus were excluded. None of the study participants had an ongoing infection during the study.

### Study Design

The study had a case–control, cross-sectional design. After obtaining written informed consent, medical history was taken, patients filled out the COPD Assessment Test (CAT) and modified Medical Research Council (mMRC) questionnaires, body plethysmography and arteriography were performed and arterialised capillary blood gases were measured. Venous blood was taken for total cholesterol, HDL-cholesterol, LDL-cholesterol, triglyceride, hsCRP, IL-6, endothelin-1 and suPAR measurements.

The study has been approved by the Semmelweis University Ethics Committee (TUKEB 131/2017), and all participants gave their informed consent.

### Body Plethysmography

Lung function tests and body plethysmography were carried out with the PDD-301/s device (Piston Ltd., Budapest, Hungary) according to the American Thoracic Society guidelines [16]. Lung function indices were calculated using the best of three technically acceptable measurements.

### Measurement of Arterial Stiffness and Blood Pressure

Blood pressure parameters and arterial stiffness parameters, including aortic pulse wave velocity (PWVao) and Aortic Augmentation Index (Aix) measurements were performed in the supine position and after 10 min of rest using an invasively validated oscillometric, upper-arm cuff automatic device based on the ‘occluded artery theory’ (Arteriograph, TensioMed Ltd., Budapest, Hungary). Details of the method and its invasive validations have been published previously [17, 18]. In brief, the device first measures the actual brachial systolic blood pressure (SBPbr) and brachial diastolic blood pressure (DBP) with a clinically validated algorithm [19]. The cuff is then inflated to a supra-systolic pressure (35–40 mmHg above the actual brachial systolic blood pressure) occluding the brachial artery completely. Pure pressure signals are collected by the cuff in this condition. The time difference between the early and late systolic peaks, is the return time (RT). By dividing the return time by 2, the transit time of the aortic pulse wave is obtained. By measuring the jugulum to symphysis straight distance between the suprasternal notch and pubic bone (an acceptable estimate of the aortic length [20]), divided by transit time, the PWVao (m/s) can be calculated. The Augmentation Index (Aix) was calculated taking the differences between amplitudes of the forward and reflected systolic waves. The left ventricle ejection duration (ED) is calculated from the pulse waves, by measuring the time between the opening and closing of the aortic valve. The Arteriograph calculates the central blood pressure (SBPao) based on the brachial SBPbr and the pulse pressure curve. SBPao is the systolic blood pressure measured at the aortic root. The difference between the central and peripheral systolic pressure (SBPao–SBPbr) is called pressure amplification. At younger ages when the aortic wall is still elastic the SBPao is less than SBPbr on the upper arm.

### Circulating Biomarkers

Plasma was isolated from EDTA anticoagulated fasting blood samples and stored at − 80 °C until measurement. Plasma suPAR concentrations were measured with the suPARnostic Flex ELISA assay (ViroGates A/S, Birkerød, Denmark) according to the manufacturers’ instructions. Interleukin-6 levels were analysed by the Immulite 2000 immunoassay system (Siemens Healthcare GmbH, Erlangen, Germany). Plasma endothelin-1 levels were determined with the Endothelin (1–21) ELISA Kit (Biomedica, Medizinprodukte GmbH & Co KG, Wien). The hsCRP levels were measured using commercially available tests (Roche Diagnostics GmbH, Mannheim, Germany). The technicians who measured the samples were blinded to the identity of the patient samples.

### Statistical Analysis

GraphPad Prism 5.03 (GraphPad Software, La Jolla, CA, US) was used for statistical analysis. Data normality has been assessed with the Shapiro–Wilk test. COPD and control groups were compared with un-paired *t* test, Mann–Whitney and Chi square tests. The relationships between plasma suPAR levels and clinical variables as well as circulating biomarkers were assessed with Pearson’s and Spearman’s tests. Data are expressed as mean ± standard deviation for parametric and median/range/for non-parametric variables. A *p* value < 0.05 was considered significant.

The sample size was calculated to find a difference between COPD and control group with an effect size of 0.90, power of 0.80 and an alpha of 0.05 [21]. These numbers were based on a distribution of plasma suPAR values [22]. Post hoc sensitivity analyses ensured it was possible to detect correlations between suPAR and clinical variables as well as other plasma biomarkers with an effect size of 0.54 (− 0.40 and 0.40, minimal and maximal critical *r* values), statistical power of 0.80 and alpha of 0.05 [21].

## Results

### Comparison of COPD and Control Groups as Well as Ever- and Never-Smoker Controls

**Fig. 2:**
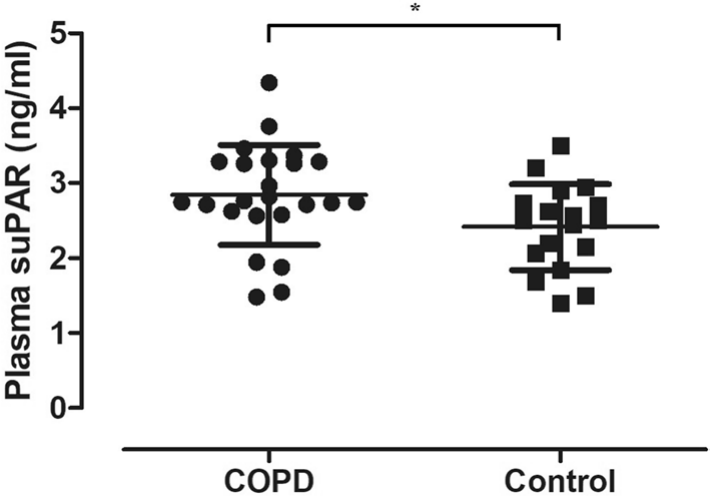
Plasma suPAR levels in COPD and controls. Significantly higher plasma suPAR levels were detected in COPD (*p = 0.03). Individual data are presented with mean ± standard deviation

Characteristics of the study population are presented in Table 1. The levels of plasma suPAR were significantly higher in patients with COPD (2.84 ± 0.67 ng/ml vs. 2.41 ± 0.57 ng/ml, p = 0.03, Fig. 2).

**Table 1:** Subjects’ characteristics

	COPD (*n* = 24)	Controls (*n* = 18)	*p* value
Age (years)	60.9 ± 5.3	58.4 ± 6.5	0.16
Gender (males%)	54%	33%	0.18
Smoker (ever/never)	23/1	9/9	< **0.01**
Smoker (current/ex/never)	9/14/1	8/1/9	< **0.01**
Cigarette pack years	33.9 ± 18.2	11.4 ± 15.2	< **0.01**
Number of frequent exacerbators	12	NA	NA
FEV_1_ (l)	1.43 ± 0.67	2.81 ± 0.67	< **0.01**
FEV_1_ (% pred.)	47.8 ± 22.4	101 ± 19.9	< **0.01**
FVC (l)	2.7 ± 0.83	3.6 ± 0.9	< **0.01**
FVC (% pred.)	69.7 ± 23.3	107.6 ± 18.2	< **0.01**
FEV_1_/FVC (%)	51.9 ± 12.7	78.2 ± 3.9	< **0.01**
RV (l)	4.2 ± 1.6	2.2 ± 0.8	< **0.01**
TLC (l)	7.3 ± 1.8	5.9 ± 1.6	0.018
RV/TLC (%)	57.3 ± 11.9	36.7 ± 8.4	< **0.01**
Raw (kPa*s/l)	0.48 ± 0.2	0.28 ± 0.1	< **0.01**
pO_2_ (mmHg)	65.1 ± 7.4	76.8 ± 8.1	< **0.01**
pCO_2_ (mmHg)	41.1 ± 4.7	38.9 ± 2.7	0.13
CAT	18.5 ± 7.2	7.8 ± 2.7	< **0.01**
mMRC	1.8 ± 0.8	0.2 ± 0.4	< **0.01**
Total cholesterol (mmol/l)	5.4 ± 0.8	5.1 ± 0.8	0.42
Triglyceride (mmol/l)	1.3 ± 1.0	1.9 ± 1.5	0.18
HDL-C (mmol/l)	1.7 ± 0.3	1.4 ± 0.25	0.04
LDL-C (mmol/l)	2.9 ± 1.0	3.0 ± 0.5	0.67
hsCRP (mg/l)	2.50 /0.50–7.80/	1.65 /0.5–4.9/	0.14
IL-6 (pg/ml)	4.29 /2.61–13.63/	3.47 /1.65–5.75/	**0.03**
suPAR (ng/ml)	2.8 ± 0.7	2.4 ± 0.6	**0.03**
ED-1 (fmol/ml)	1.3/0.0–10.1/	0.8/0.0–6.1/	0.18

Data are expressed as mean ± standard deviation or median/range/or percentage. Significant differences are highlighted in bold

*COPD* chronic obstructive pulmonary disease, *FEV*_1_ forced expiratory volume in 1 s, *FVC* forced vital capacity, *RV* residual volume, *TLC* total lung capacity, *Raw* airway resistance, *CAT* COPD Assessment Test, *mMRC* Modified Medical Research Council Dyspnea Scale, *HDL-C* high-density lipoprotein cholesterol, *LDL* low-density lipoprotein cholesterol, *hsCRP* high-sensitivity C-reactive protein, *IL-6* interleukin-6, *suPAR* soluble urokinase-type plasminogen activator receptor, *ED-1* endothelin-1

Ever-smoker controls (*n* = 9) tended to have elevated plasma suPAR levels, and significantly increased PWVao as well as decreased RT, FEV_1_ and FVC compared to never-smokers. Comparison of smoker and non-smoker controls is presented in Table 2. There was a significant correlation between cigarette pack years and plasma suPAR levels in controls (*r* = 0.68, *p* < 0.01).

**Table 2:** Comparison of ever- and never-smokers

	Control Never-smoker (*n* = 9)	Control Ever-smoker (*n* = 9)	*p* value
FEV_1_ (l)	3.1 ± 0.68	2.5 ± 0.54	**0.05**
FEV_1_ (% pred.)	103.8 ± 21.5	99.3 ± 19.9	0.28
FVC (l)	4.1 ± 0.9	3.2 ± 0.7	**0.02**
FVC (% pred.)	112.3 ± 19.1	102.9 ± 17.0	0.3
FEV_1_/FVC (%)	76.8 ± 3.8	79.6 ± 3.7	0.13
RV (l)	2.56 ± 0.9	1.9 ± 0.67	0.17
TLC (l)	6.8 ± 1.8	5.1 ± 0.8	0.11
RV/TLC (%)	37.1 ± 6.3	36.4 ± 10.22	0.74
Raw (kPa*s/l)	0.26 ± 0.8	0.31 ± 0.4	0.27
hsCRP (mg/l)	1.4/0.50–3.8/	1.9/0.7–4.9/	0.18
IL-6 (pg/ml)	3.3/2.55–4.13/	3.7/1.65–5.75/	0.29
suPAR (ng/ml)	2.1 ± 0.5	2.7 ± 0.5	0.08
ED-1 (fmol/ml)	0.29/0.0–3.1/	1.17/0.3–6.1/	0.11
SBPbr (mmHg)	129 ± 11.1	131 ± 8.4	0.76
DBP (mmHg)	78 ± 8.6	76 ± 6.4	0.71
HR (min)	67 ± 8.6	71 ± 17.8	0.52
PP (mmHg)	51.1 ± 6.9	52.0 ± 6.1	0.78
SBPao (mmHg)	122/98–142/	129/113–148/	0.71
SBPao–SBPbr (mmHg)	− 5/− 10 to 2/	− 1.1/− 11 to 7/	0.18
Aix%	− 12.2 ± 23.6	− 2.6 ± 33.0	0.49
ED (ms)	320.0 ± 25.9	321.1 ± 33.1	0.94
PWVao (m/s)	8.1 ± 0.9	9.5 ± 1.3	**0.03**
RT (ms)	127.7 ± 15.8	102.7 ± 16.3	< **0.01**

Data are expressed as mean ± standard deviation or median /range/or percentage. Significant differences are highlighted in bold

*FEV*_1_ forced expiratory volume in 1 s, *FVC* forced vital capacity, *RV* residual volume, *TLC* total lung capacity, *Raw*: airway resistance, *hsCRP* high sensitivity C-reactive protein, *IL-6* interleukin-6, *suPAR* soluble urokinase-type plasminogen activator receptor, *ED-1* endothelin-1, *SBPbr* brachial systolic blood pressure, *DBP* brachial diastolic blood pressure, *HR* heart rate, *PP* pulse pressure, *SBPao* the central blood pressure, *SBPao* SBPbr pressure amplification, *Aix* Augmentation Index, *ED* ejection duration, *PWVao* aortic pulse wave velocity, *RT* return time

### Relationship Between Circulating suPAR and Measures of COPD Severity and Activity

**Fig. 3:**
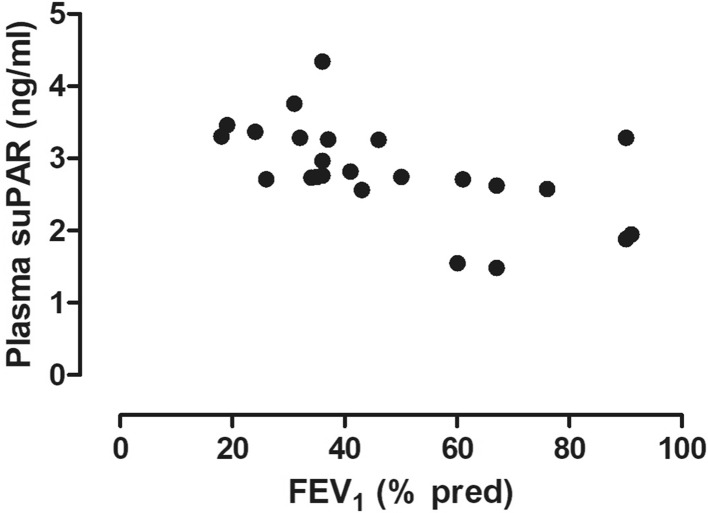
Relationship between plasma suPAR levels and lung function. A significant relationship was detected between plasma suPAR levels and FEV_1_ (*r* = − 0.65, *p* < 0.01)

**Fig. 4:**
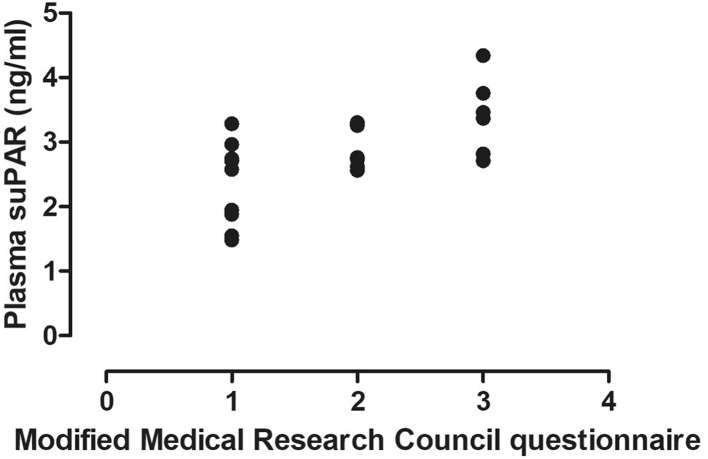
Relationship between plasma suPAR levels and symptoms burden. A significant relationship was detected between plasma suPAR levels and mMRC score (*r* = 0.55, *p* < 0.01)

There was a significant relationship between circulating suPAR levels and FEV_1_% (*r* = − 0.65, *p* < 0.01, Fig. 3), FEV_1_/FVC (*r* = − 0.46, *p* = 0.02) and mMRC (*r* = 0.55, *p* < 0.01, Fig. 4). In addition, plasma suPAR levels tended to be elevated in patients with frequent exacerbations (3.09 ± 0.39 ng/ml vs. 2.58 ± 0.79 ng/ml, *p* = 0.058). In contrast, there was no relationship between plasma suPAR levels and FVC, pO_2_, pCO_2_, *R*_aw_, RV, TLC, RV/TLC or CAT (all *p* > 0.05).

### Relationship Between Circulating suPAR and Markers of Arterial Stiffness

Comparison of the two groups in terms of arterial stiffness is found in Table 3. There was a significant difference in SBPao, SBPao–SBPbr, PWVao and RT (all *p* < 0.05), suggesting increased arterial stiffness in COPD.

**Table 3:** Comparison of measures of blood pressure and arterial stiffness between COPD and controls

	COPD (*n* = 24)	Controls (*n* = 18)	*p* value
SBPbr (mmHg)	135 ± 12.4	130 ± 9.6	0.18
DBP (mmHg)	85 ± 10.9	77 ± 7.3	**0.02**
HR (min)	72 ± 16	69 ± 13.7	0.59
PP (mmHg)	53.5 ± 10.5	51.6 ± 6.4	0.50
SBPao (mmHg)	143/106–156/	123/98–148/	< **0.01**
SBPao–SBPbr (mmHg)	4/− 16 to 11/	− 4/− 11 to 7/	< **0.01**
Aix%	10.1 ± 30.6	− 7.4 ± 20.3	0.06
ED (ms)	308.5 ± 36.5	320.6 ± 28.7	0.25
PWVao (m/s)	10.6 ± 1.9	8.8 ± 1.3	< **0.01**
RT (ms)	99.6 ± 20.6	115.2 ± 20.2	< **0.01**

Data are expressed as mean ± standard deviation or median /range/or percentage. Significant differences are highlighted in bold

*SBPbr* brachial systolic blood pressure, *DBP* brachial diastolic blood pressure, *HR* heart rate, *PP* pulse pressure, *SBPao* the central blood pressure, *SBPao*–*SBPbr* pressure amplification, *Aix* Augmentation Index, *ED* ejection duration, *PWVao* aortic pulse wave velocity, *RT* return time

When all subjects were investigated together, a significant correlation was seen between plasma suPAR concentrations and ejection duration (*r* = − 0.31, *p* = 0.04), PWVao (*r* = 0.38, *p* = 0.01) and RT (*r* = − 0.31, *p* = 0.04), but there was no correlation with Aix%, PP or SBPao (all *p* > 0.05).

When only the COPD subjects were analysed, only ejection duration correlated with plasma suPAR levels (*r* = − 0.44, *p* = 0.03).

### Relationship Between Circulating suPAR and IL-6, Endothelin and hsCRP

There was a significant direct relationship between circulating suPAR concentrations and IL-6 (*r* = 0.45, *p* < 0.01), hsCRP (*r* = 0.47, *p* < 0.01) and endothelin-1 (*r* = 0.48, *p* < 0.01) levels in all subjects. When the COPD subjects were analysed separately, plasma suPAR related to hsCRP (*r* = 0.53, *p* < 0.01) and endothelin (*r* = 0.54, *p* < 0.01) and tended to be related to IL-6 (*r* = 0.40, *p* = 0.051).

## Discussion

We investigated the plasma levels of suPAR, a novel biomarker of inflammation in COPD. We found elevated levels in COPD which correlated with lung function and symptom burden. A significant association was also found between increased suPAR levels and arterial stiffness, suggesting that this molecule may play a role in development of atherosclerosis in COPD.

The prevalence of cardiovascular comorbidities [23], including atherosclerosis [24] is high in COPD, and COPD is also prevalent in patients with known atherosclerosis [25]. Arterial stiffness is not only a marker of clinically symptomatic disease, but preclinical atherosclerosis as well. Recent meta-analysis reported impaired endothelial function, a surrogate for arterial stiffness in COPD [26]. This has been confirmed by our study reporting increased arterial stiffness in patients with COPD. The pathomechanism linking COPD to atherosclerosis is complex and includes common risk factors, such as smoking, pollution, male gender, and aging and systemic inflammation [24]. Pro-inflammatory cytokines, such as IL-6 or TNF-α are elevated in blood samples of COPD patients and can induce the release of CRP and pro-coagulant mediators by the liver, but have also a direct effect on endothelium [24]. IL-6 and TNF-α can also induce the production of suPAR from monocytes and lymphocytes [27], which has a well-established role in the development of atherosclerosis [28]. It induces cellular adhesion, leukocyte migration and eventually leads to the formation of an atherosclerotic plaque [29, 30]. Previous studies revealed that increased plasma suPAR levels were associated with risks for subclinical carotid atherosclerosis and increased occurrence of carotid plaque and cardiovascular disease [31, 32]. The prognostic value was independent from traditional risk factors (i.e., age, gender, smoking, hypertension, dyslipidemia, diabetes), and hsCRP [33, 34]. Moreover, the Monitoring Trends and Determinants in Cardiovascular Disease study compared suPAR with CRP and showed that suPAR is more related to endothelial dysfunction and atherosclerosis than CRP [35].

Circulating suPAR levels have been determined in COPD only by a few studies [12–15]. Two studies analysed suPAR during acute exacerbations [12, 13]. These events are associated with airway and systemic inflammatory responses, and not surprisingly elevated suPAR levels were found during exacerbations [12, 13]. The results in stable disease are contradictory, as both higher [14] and similar [15] levels were found. Comparing the two studies, the most striking difference was observed in the severity of airflow limitation, as Wang et al. recruited participants with milder severity [15]. However, large COPD studies, such as COPDGene or ECLIPSE confirmed a significant relationship between the extent of airflow limitation and magnitude of systemic inflammation [36]. We included patients with a wide range of lung function and found a significant relationship between airflow limitation and higher suPAR levels.

The most likely explanation of increased suPAR levels in COPD is the increase in IL-6 which is in line with the literature [37]. IL-6 upregulates suPAR production [27] which was supported by a significant association between these two molecules in our study. Circulating IL-6 induces the endothelium to release chemotactic factors for leukocytes and adhesion molecules [24], and high blood levels are associated with cardiovascular comorbidities in COPD [38]. SuPAR which is also induced by IL-6, have more direct effect in formation of the atherosclerotic plaques [29, 30], and therefore, may be a more specific biomarker of endothelial dysfunction in COPD than other circulating mediators. The clinical role of using suPAR as a biomarker for cardiovascular disease has already been assessed [11]. The current study implies that this molecule can also be useful in COPD and associated atherosclerosis, however, this has to be confirmed in larger cohorts.

Apart from lung function, suPAR was associated with symptom burden measured by the Mmrc in which score reflects breathlessness in relation to physical exercise. It is likely that this association is not independent from lung function, but due to the low number of subjects we did not test this. There was a tendency for higher plasma suPAR levels in patients with frequent exacerbations. This is in line with the findings of the ECLIPSE cohort that persistent systemic inflammation is related to the frequency of exacerbations [39]. The predictive value of suPAR to detect patients with higher risk for exacerbation has to be assessed in independent cohorts. Interestingly, we did not find any association between plasma suPAR levels and blood gases, suggesting that hypoxia may not be a strong signal for suPAR production, however, this has to be tested as well.

The biggest limitation of our study was a relatively low sample size. This was based on our previous study using the same medium (EDTA-treated plasma samples) and analytical technique [22]. This sample size allowed us to explore univariate relationships between suPAR and clinical variables, however, to conclude on independency of these association, a higher sample size is warranted. A significant relationship was found between plasma suPAR concentrations and markers of arterial stiffness when all subjects were investigated, however, many of these correlations disappeared when only the COPD patients were studied. A possible explanation could be the low sample size, but more likely it is due to the relatively mild extent of atherosclerosis in these patients as we excluded those patients with a manifesting cardiovascular disease. Ejection duration was the only variable which remained significantly associated with plasma suPAR in COPD. Increased arterial stiffness decreases systolic ejection duration which is associated with impaired coronary blood flow, therefore, ED serves as an important biomarker for development of coronary artery disease [40]. The reason why suPAR correlated with this and no other markers of arterial stiffness has to be explored in further studies.

The control groups included ever and never-smoker participants. Previous studies reported higher circulating suPAR levels in smokers [41, 42] which was confirmed in this study. There were significant differences in the prevalence of smokers and smoking history between the COPD and control groups, and ever-smoker controls had lower lung function volumes and elevated markers of arterial stiffness. The inter-group difference in suPAR may partially result from smoking. Future studies should aim for control groups with a more balanced smoking history.

## Conclusion

In summary, we reported higher plasma suPAR levels in COPD, which are associated with impaired lung function and increased arterial stiffness. Plasma suPAR may be a potential link between COPD and cardiovascular comorbidities, however, this and its potential biomarker role have to be investigated in further studies.
